# Health providers’ perspectives on delivering public health services under the contract service policy in rural China: evidence from Xinjian County

**DOI:** 10.1186/s12913-015-0739-x

**Published:** 2015-02-27

**Authors:** Huixuan Zhou, Weijun Zhang, Shengfa Zhang, Fugang Wang, You Zhong, Linni Gu, Zhiyong Qu, Xiaoyun Liang, Zhihong Sa, Xiaohua Wang, Donghua Tian

**Affiliations:** China Institute of Health, School of Social Development and Public Policy, Beijing Normal University, 19 Xinjiekou Wai Street, Haidian District, Beijing, 100875 China

**Keywords:** Village doctors, Contract service, Public health care, Health providers, China

## Abstract

**Background:**

To effectively provide public health care for rural residents, the Ministry of Health formally unveiled the contract service policy in rural China in April 2013. As the counterpart to family medicine in some developed countries, the contract service established a compact between village doctors and local governments and a service agreement between doctors and their patients. This study is a rare attempt to explore the perspectives of health providers on the contract service policy, and investigate the demand side’s attitude toward the public health services delivered under the contract policy. This evidence from Xinjian County, Jiangxi Province, the first and most representative pilot site of the contract service, could serve as a reference for policymakers to understand the initial effects of the policy, whereby they can regulate and amend some items before extending it to the whole country.

**Methods:**

Official documents were collected and semi-structured interviews with human resources and villagers in Xinjian County were conducted in September 2013. A purposive sampling method was used, and eight towns from the total 18 towns in Xinjian County were selected. Ultimately, eight managers (one in each township health center), 20 village doctors from eight clinics, and 11 villagers were interviewed. A thematic approach was used to analyze the data, which reflected the people’s experiences brought about by the implementation of the contract service policy.

**Results:**

While the contract service actually promoted the supply side to provide more public health services to the villagers and contracted patients felt satisfied with the doctor-patient relationship, most health providers complained about the heavy workload, insufficient remuneration, staff shortage, lack of official identity and ineffective performance appraisal, in addition to contempt from some villagers and supervisors after the implementation of the contract service.

**Conclusions:**

Contract service is a crucial step for the government to promote public health services in rural areas. To inspire the positive perspective and optimal work performance of the health workforce, it is imperative for the Chinese government to fortify financial support to health providers, adopt an advanced management model and escalate administrative capacity.

## Background

One key objective of China’s new health reform in 2009 was to improve the equality and dispersal of public health services. Several policies have been implemented to counter the negative aftermath of economic reform in the early 1980s, which seriously undermined the public health system, especially in the relatively poorer rural areas. During the economic reform, the central government dramatically reduced the financial investment in health care services, and the rural commune economy, as the basis of the Cooperative Medical System collapsed [1]. As a result, 900 million rural residents lost health insurance overnight, and the barefoot doctors in village clinics became unemployed. To make a living, they turned to provide medical services and sell drugs as private practitioners and naturally gave up public health services without any financial support from the government. Other public health facilities also focused on the sales of medical services for profit to offset the decline in public expenditure on health. In the meantime, the responsibility to support health services was adopted by the local governments, and the disparity of economic status between the urban and rural areas largely impaired the health equality [2]. Unlike the richer urban population who enjoyed relatively complete health care financed by their local governments, the poor, especially the elderly, in rural areas suffered from heavy disease burden [3,4]. The gap in life expectancy between the richest municipality and poorest province of China was 13 years in 2000 [5]. The gap in the USA was 6.5 years in 2001 [6] and 2.5 years in the UK in 2004 [7]. Therefore, the establishment of the New Cooperative Medical System (NCMS) was the foremost policy in the new round health reform [8], which covered health insurance for the rural population and underpinned the revival of rural health services.

Meanwhile, the central government instituted basic items of public health services, including the establishment of citizens’ health assessments, health education, vaccine inoculation and immunization, health protection of children, maternity patients, and elderly, chronic diseases control including hypertension, type II diabetes, and severe psychosis, response to public health emergencies, and sanitary supervision [9]. The township health centers (THCs) and village clinics were obligated to provide these items and were financed by public health funding, which was raised by the central and local governments. However, most village clinics have been privatized for 30 years, and the awareness of public health might have faded from most health providers’ minds over such a long time. Consequently, the Ministry of Health formally unveiled the contract service policy in April 2013 to assist the organization work of public health services and change the village doctors’ work emphasis from curative treatment into primary health care. The Ministry planned to implement this policy in several pilot sites, and then extend it all over the country in 2014 [10].

The contract service in rural China can be referred to as the counterpart to family medicine in some developed countries [11-13]. It can be defined as contracts endorsed between village doctors and rural citizens in the household units, which facilitate the doctors in providing more demand-tailored public health services like general practitioners. In addition, it is a contract signed between THCs and village doctors. The THCs are in the charge of public health funding, and are obligated to cooperate with village clinics and supervise the behavior of the village doctors under their jurisdiction.

The level of public health funding has been raised annually. The governments allocated ¥25 (US $4.00) for each person of service population in 2012 and ¥30 (US $4.80) in 2013. According to the official regulation, the THCs are required to allocate no less than 40% of the money to village clinics. In general, this part of the funding is divided into two parts. The first part, which accounts for approximately 70% of the funding, is distributed to the village doctors regularly. The remainder is assigned by the THCs to the village doctors on the basis of performance appraisal of public health practice. Although the public health services should be provided whether the contracts exist or not, the Ministry intended that the procedures of signing the contracts could make both the demand and supply sides understand their rights and duties in public health more explicitly and could promote health providers to commit themselves to public health services under an effective management and supervision system.

The delivery of public health care under the contract service provides the rural citizens with free public health services without imposing any restrictions on them, except for following the doctor’s advice. In contrast, it assigns complicated service items to the local health providers and establish a new relationship between the village clinics and THCs. The implementation of this policy would make some changes to health providers, and they would have some perspectives on it. Awareness of their thoughts is very important for the policymakers because health workers are the crucial stakeholders in any reform process. The contract service could not work smoothly without their positive cooperation, let alone be extended to the whole country. However, research into the perspectives of the supply side on the contract service has been seldom conducted in rural health systems. The likely main reason for this problem is that the contract service policy has not been implemented experimentally for a long time and the effects of this policy are not visible. Although the Ministry of Health selected 15 counties as pilot sites (one in each province), most of them had applied this policy for only a few months before our investigation. Fortunately, there are a couple of pilot counties where the contract program has already in place since 2012. Xinjian County, Jiangxi Province is the earliest one among the pilot sites, which was the most important motivation for us to choose it as the study site.

The purpose of this study is to explore the perspectives of the rural health providers (including both THC staff and village doctors) on delivering public health services under the contract service policy, and investigate some demanders’ attitudes toward the contract service as a complement. This study could serve as a reference for the policymakers to understand the real circumstances of the policy implementation, whereby they can regulate and amend some items before extending it to the whole country. This qualitative study is suitable to explore people’s experiences and their direct feedback to changes happening with them and can be an exploration for further investigations on the contract service.

### Contract service in Xinjian County

In addition to being the first experimental site of the contract service from August 2012, Xinjian County was selected as the study site for some other reasons. First, Xinjian is a typical agricultural county with a total population of 686,800, of whom 79% represent the agricultural population. Second, the coverage of health insurance, namely the NCMS, reached 99.99% in 2013, which was regarded as a solid cornerstone for the contract policy and public health services. Third, Xinjian’s economy ranks in the middle range of China. The per capita Gross Domestic Product (GDP) was ¥44925 (US $7241) in 2013, which was close to the national average of ¥41027 (US $6629) [14]. Furthermore, Jiangxi Province is a midland area located in the middle of the Chinese mainland, and its economic development is approximately in the middle level for China. Fourth, the contract service policy has covered the whole county, and its mode of implementation fairly complies with the governmental regulation. In general, Xinjian, a county with a modest background is suitable to be the study site in many aspects.

## Methods

Qualitative methods were applied in this study because they were adept in answering “how” and “why” questions [15]. We collected official documents related to the contract services in Xinjian County and intended to extract some data or information from them. Moreover, the semi-structured interview with human resources and villagers in Xinjian County were conducted over six months beginning in September 2013.

### Sampling

Eight towns from the total eighteen towns in Xinjian were selected by the purposive sampling method. First, two towns exemplary in service quality were chosen in our study. Second, considering that the terrain of Xinjian County is long and narrow from north to south, we selected six towns with heterogeneity in economic development and health service quality in the northern and southern areas. Every town had one township health center, and one manager of each THC was selected to participate in the interview. Then, one village clinic in each town was selected by random sampling, and all doctors in that clinic were invited to join the interview. Patients or voluntary villagers were interviewed conveniently in the village clinics. The study protocol was approved by the Ethical Review Board of Beijing Normal University [16].

### Data collection

The outline of semi-structured interview for health providers was derived from framework in terms of job satisfaction theory [17-21]. In addition to some questions about personal information, core questions related to changes brought about by the contract service in aspects such as income, trainings and promotions, models of administration, working hours and relationships were asked. Lastly, we set up some open questions about pre- and post-comparisons in other aspects and about suggestions for future work. The interview for villagers focused on their knowledge and attitudes about the contract service and their behaviors in seeking medical care; it was simpler than the version for providers because villagers signed the contract voluntarily without imposing obligations on them and our study was mainly concerned about changes happening with the supply side.

Examples of questions to village doctors:

1. What changes have taken place in work content after signing the contract? Is there any change in working hours? What do you think about your current workload?
2. What changes have taken place in the doctor-patient relationship after signing the contract? What do the villagers think about the contract? Are they willing to cooperate with your work?

Examples of questions to THC managers:

3. How do you assign work task of public health services? Which service items are taken by THC? What changes have taken place in the workload after signing the contract?
4. Can the public health funding offset your operating cost? What changes have taken place in your staff’s income after signing the contract? Do they feel satisfied with it? If not, would you tell me the reasons?

Example of questions to villagers:

5. Have you ever heard of contract service? Do you sign the contract and do you know the content of public health services?
6. What changes have taken place in the health care you received after signing the contract? Do they feel satisfied with it? If so, would you give me some details? If not, would you give me some suggestions?

Verbal consent was taken from all interviewees instead of signing informed consent because they had concerns and misgivings about the interviews. Interviews with THC staff were conducted in their offices, and those with village doctors were conducted in their homes to facilitate open and free discussion by a sense of security, especially when people intended to discuss some sensitive topics. Interviews lasting for 30 to 60 min were tape-recorded and transcribed verbatim without any mark of the interviewees’ names and accurate addresses to avoid the divulging of their information. Likewise, the quotations in the Results section only mention the interviewees’ posts.

### Data analysis

Based on the thematic analysis approach [22], the authors examined the transcripts independently with open coding. First, we sorted out these codes according to themes manifested in former research and the factors commonly mentioned by the interviewees. Second, we generated inductive code, allowing the raw data to be summarized by our categories. In addition, we ignored the concepts which had little relationship with the topics we were concerned and clarified the interaction between inductive codes [23]. Finally, we made some causal inferences pertaining to the policies of health reform. This process allowed the authors to identify the scope and relationships between concepts that grounded the key interpretations and dimensions of the research questions [15].

## Results

In total, we interviewed eight THC managers, 20 village doctors from eight clinics and eleven villagers. The total service population of the eight THCs was 236,235. The village doctor interviewees were all males, and twelve of them were over 50 years old and only one was a licensed assistant doctor. Through coming into contact with 28 health workers and eleven villagers in Xinjian, we believed that the data collection achieved “data saturation” due to the repetition of interviewees’ perspectives. The data narrated in the following six aspects did reflect people’s experiences brought about by the implementation of the contract service policy.

### Workload

In contrast to the old days when they mainly took responsibility of the medical services for local residents, all of the village doctors thought that their work burden had increased by providing public health care and primary prevention after the contract service was implemented. Everyday medical care had already taken up most of their working hours. Each of them had the experience of working overtime for public health care, ranging from two hours to eight hours a day. The average service population per village doctor in Xinjian County was 809, which exceeds the upper limit of 600 recommended by the Ministry of Health for a qualified general practitioner. In the terms of unlicensed village doctors, the view was that an appropriate service population per doctor should be no more than 300. Village doctors were confronted with tremendous work pressure, especially in the clinics with less doctor distribution.

“I often didn’t have enough time to sleep because I dealt with too many patients during the day, and then, I have to follow up with the chronic cases in the evening. As soon as I get home, I find that there is a lot of information that must be inputted into the computer. I often go to bed after midnight since the contract policy was implemented. As you know, there are 1,700 residents in our village, and my partner died months ago, but no one has come to fill the vacancy. I have to treat all of them by myself”. (Village doctor)

Village doctors had to offer a health assessment to every villager, manage and follow up on patients with chronic diseases and input the related data into computers as part of the performance appraisal items. It was difficult for most of them to operate an information system on the computer given their old ages and simple education backgrounds. They needed to receive skilled training in computer operation.

“I am already 56 years old, and I am the only doctor in our village……I am not accustomed to using computers and always ask my children for help.” (Village doctor)

Not only did the village doctors complain about their heavy workload, but the THC managers also argued that the administrative affairs occupied their time. Village clinics had no equipment or adequately qualified workforce to undertake the villagers’ physical examinations, inoculations, child and maternity protection and diagnosis of most chronic diseases. Therefore, the organization work and most public health services were the responsibilities of the THCs.

“We arrange medical cadres with equipment to assist village doctors many times a year. Meanwhile, implementing propaganda on public health to the villagers and organizing health education for the patients also fall under our duties”. (THC Manager)

“Most village doctors cannot pass the qualifying examinations, so we organize monthly training activities and helped them to promote health knowledge and service ability. In addition, they had to supervise the doctors’ behaviors and work achievements by randomly calling villagers to ask whether their doctor provided public health care properly and to verify the computer records of health assessments”. (THC Manager)

According to the collected documents, public health services in Xinjian County were fully under way until 2011, and two years afterward the basic items of public health were instituted by the central government. In spite of the long term preparations, only 10.45% of rural citizens had their health assessment archives recorded in the computer system. This proportion had dramatically increased to 60.19% in February 2013, less than six months after the contract service policy was implemented. Combined with the information extracted from interviews, it indicated that the public health services represented only a slogan without the contract signing program. Village doctors did not engage themselves into providing public health care until they recognized during the contract procedure that they could receive a certain proportion of public health funding if they provided the public health product. They had been private practitioners and independent of governmental subsidy for decades. The contract and the remuneration related to public health attracted them to cooperate with THCs. Naturally, THCs, as their superior departments and the managers of funding, began to supervise and appraise their work achievements. When they settled down to the genuine work, several problems emerged. The contract service triggered workload explosions to both doctors and managers and reflected the lack of and the aging of the workforce. Meanwhile, insufficient remuneration was another important reason for their complaints about the contract service.

### Remuneration insufficiency

All providers implied that their heavy workload demanded more than what they had gained. The structure of the village doctors’ income had gradually changed from medication fees to a more complex constitution composed of registration fees, compensation for selling essential drugs with zero markup, and subsidies for the public health service since 2009. The annual earnings of the interviewed village doctors ranged from ¥10,000 (US $1607) to ¥28,000 (US $4500). The annual subsidy for public health per village doctor ranged from ¥4,000 (US $642) to ¥8,000 (US $1286) according to the service population in their villages. The contract service took up so much time of the village doctors that it even limited their performance in medical care, while the payment related to public health was disproportional to their workload. Although their incomes were higher than the net per capita income of a rural resident in Xinjian (¥7785 or US $1249), they were much less than that of a factory labor or a migrant worker, who could earn approximately ¥60,000 (US $9642) per year. Therefore, it was common for village doctors to leave their posts to become migrant workers, and their colleagues, who actually provided the service in the clinics, had to share the remuneration equally among them because the remuneration was distributed to the doctors in the clinic unit by the THCs.

“I won a certification of merit in our province for my outstanding work quality (many honorary credentials are attached on the wall of the consulting room), and I consistently handle my cases with great care. However, after the contract service, I have to see patients and deal with information from day to night, while sharing the pitiful public health payment with my colleague who is in outside employment and never comes here. I feel so hurt (tearing). It is unfair!” (Village doctor)

On the THC side, managers also had their own opinions. The annual earnings of THC staff were composed of a fixed salary allocated by the government and a bonus with small disparity. The income per month of approximately ¥2,600 (US $417) was less than that of some village doctors in clinics with large service populations. All of them thought that their commitment was not valued by the government. The public health funding was considerably consumed as the operating cost. The staff hardly had incentives to provide public health care due to the low income.

“Village doctors at least receive the subsidy for public health after signing the contract, but our staff only earn a fixed salary. For the sake of funding constraints, public health funding is withheld from distribution to our staff and is used to purchase equipment and public health related consumptive materials, such as posters and leaflets”. (THC Manager)

### Assessment of performance

The standards of the assessment of performance should be clear-cut and hierarchical through the measurements of work quantity and quality. However, there were no guidelines that could be followed by the THCs to allow accurate evaluation of the performance. Every village doctor had the experience that their performance related income was forfeited in the assessment at the end of year. They wanted to know the reason and demanded transparency in the evaluation process.

“One hundred yuan was deducted from my performance related payment last year, while I didn’t know the reason. They (managers in the THC) only told me that my score of public health appraisal was less than 90”. (Village doctor)

Ambiguity in the work quantity evaluation and service quality appraisal could also be found when there were three or four doctors working together at the same clinic in some large villages. The elder doctors often took accountability for most treatment works for the sake of villagers’ preferences. However, in the absence of any effective measurement, they always shared the payment equally regardless of their de facto work performance.

“I always choose him as my doctor (pointing at the oldest doctor in the clinic). I think that he is very experienced”. (Villager)

“The younger doctor in our clinic is mainly in charge of inputting patient information…… We always divide the payment and subsidy equally”. (Village doctor)

The managers in the THCs agreed with the statement that the appraisal on village doctors was not exerted appropriately. Their explanation was that they were impeded to provide transparent and efficacious supervision by the heavy workload. They also admitted that there was no effective way to discriminate in the rewards and punishment of the village doctors, as well as themselves. The ineffective appraisal could not create any significant incentives to promote the providers’ performance, but it did frustrate their work enthusiasm.

“The distinction between the highest and lowest performance-related payment is approximately ¥100 (US $16) to ¥200 (US $32) per month. No one can gain incentives from such a little disparity”. (THC Manager)

### Identity

The identity as a civil servant (called “bianzhi” in Chinese) attached with wages, subsidies, endowment insurance and other welfare, is quite significant to people who work for the government under the human resource administration systems of China. All village doctors worried about their welfare, especially the endowment insurance, because they were still farmers and not involved in the official system with the identity of a civil servant, even though they had already signed the contract.

“The veterinarians in our village have become official governmental members for several years. However, as a doctor, I have not been given the official identity after signing the contract.” (Village doctor)

Village doctors were still farmers who did farming work in their spare time and made their fortune from selling agricultural products or other businesses [24]. However, the contract service demanded the village doctors spend much more time on health care activities than before. Their income was supposed to mainly rely on their work as a doctor instead of a farmer. Therefore, they were eager to ask for the official identity and gain the pension after they signed the contract.

“If I retire at this time, my income will be ¥65 (US $10) per month, and I cannot make a living on it”. (Village doctor)

The THCs also had an inadequate amount of identity. For the intolerable low income and heavy workload, some talented cadres resigned and took their official identity to their new workplaces along with their wages and welfare subsidized by the government. To meet the routine work demand, every THC had the experience of employing temporary workers to replenish the manpower. These workers earned lower incomes than official workers and could not enjoy the state subsidies. Consequently, the low income and heavy workload forced people to quit their jobs, and then the lack of manpower resulted in a heavier workload, which propelled workers in both official and temporary placements to leave their posts. All of the above took the shape of a vicious circle and baffled the development of human resources in the primary health facilities in rural China.

### Staff shortage

Managers and village doctors suggested that the staff numbers were always insufficient, and the heavy workload of the contract service made staff shortages more salient than ever before. In response to the low salaries and working without identity, some health providers sought other work to earn money. The rest who were too old to be migrant workers or not qualified to open private clinics deteriorated the aging and unqualified practice of the village doctor. The rural health system in Xinjian has been confronted with severe talent shortages, which had negative impacts on the staff’s performance and service quality.

“More and more village doctors have quit their jobs…… Villagers there have to go to other village clinics in the vicinity to see a doctor”. (THC Manager)

“My colleague, who holds the certification, has already gone out and opened a private clinic to make money. I have to stay here and take care of my old sick wife. I always feel exhausted, and I have to support my family with this meager income. I will continue to stick with the post until the endowment problem is solved (tearing)”. (Village doctor)

Additionally, managers had their own views on the issue of workforce shortages, and they thought that the retention and recruitment of the workforce was not optimistic in the future. To summarize their opinions, the first reason was that the government requires village doctors to pass qualifying examinations, but as most doctors could not reach the standards, they quit. Second, the remuneration did not value these health providers. People could not obtain work incentives and exert their talents as a doctor in the public health facilities. In addition, the gap between the cities and countryside has been widening steadily since the Chinese economic reform in 1978. Young people preferred living in metropolises over staying and working in the countryside. It was difficult to recruit fresh blood into the rural health facilities.

### Social relationships

Although the staff shortage and unfair low income accompanied with the heavy workload made providers feel unable to perform perfectly, the contract service was a real gratification from the point of view of patients. The interviewed villagers were patients met in the clinics or their family dependents. Their average age was 65 and all of them had signed the contract. Except for a 22-year old girl who saw a doctor to get some drugs for her toothache, the other 10 villagers reported more satisfactory relationships with village doctors than before. When signing the contract, they became aware of the public health services they deserved. Items, such as home visits, health assessments and follow-ups provided by the contractors, brought villagers much convenience.

“My father is 83 years old. He never saw a doctor throughout his life due to poverty. After signing the contract last year, the doctor came to my home and conducted a physical examination for my father. He was moved to tears at that moment for the considerate service”. (Villager)

“When my doctor visited me at home to assess my health status and establish a health archive for me, I was diagnosed with diabetes. Thanks to the contract service, I received early detection and treatment”. (Villager)

THC managers admitted that the village doctors should be regarded as the indispensable workforce in public health services because they were familiar with villagers and could easily gain trust around patients. However, sometimes the village doctors felt that their work was not respected or recognized by some villagers especially the people with few demand for health services. They did not think they failed to enjoy their welfare when they were absent from public health services such as health assessments and health education, because the funding of public health was raised by the governments rather than out-of-pocket.

“I noticed that some villagers even threw out the contract paper as soon as they left the clinic. When they became sick and asked for service items, we had to sign contract with them again”. (Village doctor)

“I signed a contract, but I have no idea about it and don’t care it at all. I am young and strong. Health protection has no business with me”. (Villager)

Some managers also indicated that some patients raised unreasonable demands and complained to THC managers about the doctors who did not meet their needs. The records of complaint would not only influence the year-end appraisal score but also reduced the doctors’ performance-related payment.

“When the village doctors are busy with their farming in the harvest season, some patients demand to see the doctor immediately. If they do not find the doctor in the clinic, or if the doctors have no time for a home visit, they will telephone us to complain about the doctor’s absence from work. I know that most complainants are not actually urgent cases or bed-ridden, but according to the appraisal standards, I have to urge the doctors to act”. (THC Manager)

On the other hand, there were something subtle in the relationship between village doctors and managers. Theoretically, the THCs and village clinics are cooperative partners in accomplishing the public health practices. However, insufficient remuneration has forced both involved parties into a competitive relationship. THC managers simultaneously represent both the “athletes” and “referees” in a sport meet based on their behaviors in providing public health care and evaluating village doctors’ performance. Three village doctors implied that the THCs covertly skimped on their subsidy by impartial work performance appraisal.

“None of village clinics received 40% of public health funding last year. The money deducted from performance related payment were kept by THCs. I think the appraisal was not reasonable to some degree”. (Village doctor)

According to the collected documents, THCs approximately allocated ¥8 for each person of service population to village clinics as their subsidies for public health in 2013, 32% of the public health funding. The money deducted from the clinics of poor work performance were not given to the superior ones. One of THC managers made clear that their staff were dissatisfied with the funding allocation.

## Discussion

This study is the first of its kind to investigate the perspectives of the supply side on delivering public health care under the contract service. Different from most preceding studies that only focused on village doctors, our study places emphasis on the THC managers as well. It is somewhat surprising in our study that the managers were also dissatisfied with their work conditions. While most health providers in this study complained about the heavy workload, insufficient remuneration and overlook or contempt exhibited by patients and supervisors, the contract actually facilitated them to provide villagers with more public health service and forced them to begin to commit to primary health care. Meanwhile, the villagers who had received the contract services felt satisfied with the doctor-patient relationship. In developed countries, general practitioners were commonly inclined to consider that they had the overall responsibility for the patients’ primary health care in a capitation-based contract [25]. Although rural contractors in Xinjian might not realize that they were endued with such earnest responsibilities, the policy would guide them along this orientation. Moreover, the contract made village doctors cooperate with the THCs in providing public health care and imparted villagers their right to enjoy free public health. The contract service made a good start in the coordination between the groups on the supply side and the demand side, which is significant in the field of public health [26].

However, the health providers’ negative perspectives on the contract services and their work circumstances might discourage the sustainable development of public health care in the long run. First, the quality of the health care service depends on health providers in sufficient numbers [27]. An unstable workforce is not conducive for the development of primary healthcare sectors [28]. Countries with low and middle incomes are confronted with human resource challenges in health care [29,30], including shortages of qualified staff [31], poor working incentives and staff absenteeism [32-34]. The contract service in Xinjian was challenged by these same problems. The ratio of physicians per 1000 persons was 1.34 in Xinjian County, 1.48 in rural China in 2013. The ratio of physicians per 1000 persons in mainland China was 2.04 in 2013 [14]. Although there was no consensus on the standard of physician density, it could be compared with the USA and UK where health services have not been in overproduction. The ratio of physicians per 1000 persons in the USA was 2.5 in 2011. In 2012, the ratio of physicians per 1000 persons in the UK was 2.8 [35].

Second, according to our former study of five counties [16], remuneration as a crucial work incentive for village doctors averaged ¥21,810 (US $3501) per year, which was much higher than the net per capita income of the rural residents at ¥6,977 (US $1120) in 2011 [36]. However, the findings in Xinjian County indicated that although their income was higher than the net per capita income of the rural residents, the village doctors preferred to compare their income with that of migrant workers. Thus, they were discontented with the status quo and intended to change their jobs into more lucrative fields. After all, they had committed most of their time to providing health services. It was reasonable that they regarded themselves as more skilled personnel than peasants.

Moreover, two studies regarding the village doctors’ perspectives on public health in two counties each in Hubei and Jiangxi provinces [37] and a county in Jiangsu province [38] indicated that they considered public health services too heavy for their manpower, the subsidy insufficient and social security absent. According to the findings in Xinjian, the contract policy had not relieved these problems yet but had increased the health providers’ burden of work.

Therefore, it could be inferred that if the contract service policy were extended, the health providers in most rural areas would likely suffer from heavy workloads, shortages of workforce, lack of identity and social security and complex social relations, which reflects the nationwide insufficient government expenditure on public health, thread bare human resource administration systems and incapacity of managing performance-related payment. Unlike the health workforce in developed countries where job satisfaction and retention mainly rest on professional guidance and trainings, harmonious relationships with colleagues and patients, family factors and vocational promotions [39-42], the health providers of rural China still focused on financial support and social security. Without sufficient financial and management investment from the governments, the retention of the workforce and quality public health services is not sustainable.

The financial investment in public health from the government was inadequate to ensure the smooth operation of the contract service. Public health care characterized by “public goods” should be supported by governmental finance. The current under-provision of public health services in China mainly resulted by the lack of government funding since the financial reform was applied in the 1980s [43]. According to the international experience, the governmental investment in public health should be no less than 1.5% of the GDP. However, this proportion in China was approximately 0.75% in 2009 [44]. The contract service imposed the workload of public health care on health providers without adequate financial support. Their dissatisfaction with the remuneration led to job abandonment or absenteeism, severe staff shortage, and ultimately failure in providing effective public health care.

Some explanations for the insufficient governmental investment in supply side of the health system are described as follows. One reason is that the government tends to support the demander instead of the suppliers [45]. The other explanation is that the government favors infectious diseases control rather than the other items of public health services [46]. In addition, our study suggested that the governments preferred subsidizing village doctors to the THC staff. However, the above interpretations partially explain the reasons behind the financial barriers. The primary cause is the absolutely inadequate financial support from the government. Health providers, including village doctors and THC staff, need more financial support to realize human resource retention and effective work incentives. The increase of funding will largely depend on the constant development of local economics in the future, because government expenditure on health is mainly determined by local fiscal capacity [47] under the highly financial decentralization. Provincial governments should be the key financing resources because they have significant autonomy in transferring resources to county and township governments, who take the main responsibility for financing health services [48].

Next, the human resource administration in China has always been bound hand and foot by the official identity. The official identity together with remuneration and benefits from the governments were bound to the worker himself instead of to the specific occupation, which means that the THCs could not supply adequate official posts to the new staff because the resigned worker took away their identity. This administrative system is not fit for great fluidity even at Xinjian County. Likewise, no more identities could be awarded to the village doctors. They were subject to an absence of pension and other social security, which not only had a negative impact on their income but also made them feel unrecognized by the government. Our findings suggested that the personnel system should be improved, which is a huge project not confined to the rural health system but also over the whole country.

Additionally, some policies regarding the human resources in health care that have been successfully implemented in developed countries might not be applicable in countries with low and middle incomes [49] due to the limited capacity to manage such policies. Considering Xinjian as an example, the appraisal for the work performance was conducted ineffectively given the staff shortage and lack of work incentives. The standards of appraisal appeared so obscure that they severely frustrated the staff’s motivation. On the other hand, compared with fee-for-service payments, doctors who were paid by the capitation fee gained more satisfaction from the demander but provided a smaller quantity of service [50]. Thus, it is important to use performance-related payment to motivate health providers, and policymakers should place emphasis on formulating logical standards for the appraisal and promoting ability to manage contract services.

On the demand side, while some patients enjoyed these public health services paid by the governments, some villagers did not pay attention to these free services and did not value the contracting paper according to the information from the interviews. It could be inferred that while the contract policy might be a proper way to provide public health care to the rural population, the intensity of the propaganda on the significance of public health and prevention should be enhanced. Health providers still needed to make further efforts in the communication with rural residents. Additionally, auxiliary measures to limit the villagers’ behaviors after they signed the contract were required.

This study has a couple of limitations that warrant mentioning. Only a precursory investigation into the demand side was conducted in our study. The convenience sampling method had selection bias. All interviewed villagers were encountered by us when they went to village clinics. That is to say, villagers who doubted or were dissatisfied with the village doctors’ services had little chance to be included in the survey. Moreover, the simple size of villagers was too small to determine a fair assessment regarding their attitudes toward the contract service. A further study would focus on the perspectives and attitudes of the demand side toward the contract service, whereby evaluating the effectiveness of this policy. Second, our study just focused on one county of the 15 pilot sites. Although, Xinjian would be a representative sample because of its economic status and health development ranking in the middle range of China and its typical characteristics as a rural county, future investigations will require embracing more sites.

## Conclusions

This study has displayed findings related to the health providers’ experience of delivering public health under the innovative contract service policy on the ground floor of rural health systems in Xinjian County, a theme that has not been investigated much in the region. The contract service is a crucial step for the government to merge the village clinics and THCs into a cohesive unit and to promote public health care in rural China. However, village doctors and THC staff, as the most essential stakeholders in the rural health system, have held negative attitudes toward the sustainable development of quality public health services because the contract increased their workload of public health care without bringing them reasonable remuneration. The village doctors have been regarded more as peasants than as doctors and still lack official identity and social security even though they signed the contract with the THCs. These work circumstances aggregated the workforce shortage and frustrated health providers in supplying quality public health services. Thus, it is imperative for the Chinese government to fortify the financial support to health providers, adopt an advanced management model and escalate administrative capacity, thereby inspiring the positive perspective and optimal work performance of the health workforce. In addition, the results can assist policymakers in amending the contract service policy before extending it to the whole country and to give political support to the rural health professionals by providing them with work incentives and retaining the workforce in the countryside in the future.

## Abbreviations

NCMS: New cooperative medical scheme
THCs: Township Health Centers
GDP: Gross domestic product
